# Role of TGF-Beta1/SMAD2/3 Pathway in Retinal Outer Deep Vascular Plexus and Photoreceptor Damage in Rat 50/10 Oxygen-Induced Retinopathy

**DOI:** 10.1155/2019/4072319

**Published:** 2019-05-22

**Authors:** Huijuan Li, Ruyuan Zhu, Ruibin Zhao, Lijuan Qian, Li Jiang

**Affiliations:** ^1^Medical School, Southeast University, Nanjing 210009, Jiangsu Province, China; ^2^Department of Pediatrics, Zhongda Hospital Affiliated to Southeast University, Nanjing 210009, Jiangsu Province, China; ^3^Jinling Hospital, Nanjing University, School of Medicine, Nanjing 210002, Jiangsu Province, China

## Abstract

In retinopathy of prematurity (ROP), outer deep vascular plexus (oDVP) was the emerging field, and the mechanisms of photoreceptor dysfunction remained to be explored. ODVP and photoreceptors were related, with oDVP being part of the supplier of oxygen and nutrients to photoreceptors, while their possible relationship in ROP was not clear. TGF-beta1 has been reported indispensable in oDVP development and altered in ROP patients and animal models. We hypothesized that the TGF-beta1 alteration in rat 50/10 oxygen-induced retinopathy (OIR) model contributed to oDVP malformation and exerted consequent effects on photoreceptor development. We first explored the profile of oDVP development in rat after birth and compared the expression of TGF-beta1 and pSMAD2/3 in Normoxia and OIR groups. Afterwards, the inhibitor of the pathway, LY364947, was used to establish the OIR, OIR+LY364947, Normoxia, and Normoxia+LY364947 groups. The oDVP and photoreceptor were examined by Isolectin B4 staining, western-blot of CD31 and Rho, and electron microscopy. ODVP sprouted at postnatal day 10 (D10) and reached the edge of retina at D14. The TGF-beta1/SMAD2/3 pathway was compromised during the critical period of oDVP development. The inhibitor simulated the oDVP retardation, pericyte, and photoreceptor malformation in the Normoxia+LY364947 group and might further compromise the development of oDVP and photoreceptor in the OIR+LY364947 group. The inhibition of the TGF-beta1/SMAD2/3 pathway indicated its critical role in oDVP malformation and photoreceptor damage, suggesting a possible therapeutic target of ROP treatment.

## 1. Introduction

Retinopathy of prematurity (ROP) is considered a disease of abnormal retinal vascularization, caused by adverse events, such as hyperoxia, hypoxia, inflammation, and malnutrition [1]. Most studies focused on vascular pathologies, including growth retardation and abnormal neovascularization, but little attention has been drawn to neuron damage. Regarding neuron damage, the rod photoreceptor has been confirmed vulnerable and its malformation and malfunction persist years after ROP resolution [2–6]. Only a few studies have explored the mechanisms underlying photoreceptor damage [7]. Photoreceptor located in the outer nuclear layer (ONL), and the ONL was partly supported by the outer deep vascular plexus (oDVP). The relationship between photoreceptor and oDVP has been explored in diabetic retinopathy (DR). Clinical data has confirmed the contributing role of oDVP nonperfusion in photoreceptor damage in DR [8–10], but their possible relationship has not been explored in ROP.

TGF-beta1 has been reported important in retinal DVP development and its up/downregulation may therefore participate in DVP malformation [11]. Its depletion resulted in oDVP disappearance in the knockdown mouse model, indicating its indispensable role in oDVP development [12]. In addition, TGF-beta1 has been reported overexpressed in the mouse OIR model and other hyperoxia-induced animal models, indicating its sensitivity to oxygen alteration [13]. Those studies implied the possible role of TGF-beta1 in the rat 50/10 oxygen-induced retinopathy (OIR) model, in which the oxygen level shifted between 50% and 10%. The rat 50/10 oxygen-induced retinopathy (OIR) model is a classic animal model in ROP research. The cycle alteration between 50% and 10% oxygen simulated the hyperoxia/hypoxia stimulation which the preterm babies endured.

TGF-beta subfamily (TGF-beta1-3, Activin, and Nodal) activates SMAD2 and SMAD3. There are five type II receptors and seven type I receptors (ALK1-7). Among those ligands, TGF-beta1–3, Activin, and Nodal lead to the activation of ALK5, ALK4, and ALK7, respectively. Namely, TGF-beta1-3 activates ALK5, which belongs to TGF*β*R1 [14]. LY364947 is a small molecule inhibitor targeting ALK5, while SB431542 is a widely used inhibitor for the TGF-beta pathway which blocks several ALKs, including ALK4, ALK5, and ALK7 [15]. In order to block the TGF-beta1/ALK5/SMAD2/3 specifically, we chose LY364947 as the tool in studying TGF-beta1/ALK5/SMAD2/3.

In this study, we established the rat 50/10 oxygen-induced retinopathy (OIR) model, examined the expression of TGF-beta1, and explored its effects on oDVP and rod photoreceptor using LY364947. The study helped us in analyzing the function of the pathway in oDVP development and contributing role in photoreceptor damage in ROP.

## 2. Materials and Methods

### 2.1. Rat 50/10 OIR Model Establishment and Inhibitor Administration

The animal study was conducted in compliance with the Guide for the Care and Use of Laboratory Animals, Southeast University (SEU). The study was approved by the Research Ethics Committee of SEU. Sprague-Dawley rats were raised for model establishment. All dams littered spontaneously, and the dams and pups were randomly divided into four groups, namely, the OIR, OIR+LY364947, Normoxia, and Normoxia+LY364947 groups. The day of birth was defined as Day 0 (D0). Shortly after birth, litters and dams of the OIR and OIR+LY364947 groups were transferred to an oxygen-controlled chamber in which the oxygen shifted between 50% and 10% every other day for 14 consecutive days. LY364947 was administered intraperitoneally every day from D10 to D14, and the dosage was 1 ng/mg [16–18]. The vehicle (0.9% saline) was given as control. At D2, 4, 6, 8, 10, 12, and 14, the pups and matched controls were humanely sacrificed by an intraperitoneal injection of 0.06 mg/g ketamine and 0.018 mg/g xylazine.

### 2.2. Isolectin B4 and NG2 Staining

After the pups were humanly sacrificed, the eyeballs were harvested. Retinas were acquired according to previous study [19]. Fixation and dehydration were accomplished with 4% paraformaldehyde (PFA) and 2×phosphate-buffered saline (PBS), respectively. The retina was acquired by discarding the anterior and posterior segments. Before staining, the retina was incubated with methanol and stored at −20°C to increase permeabilization. Staining was conducted according to the manufacturer's protocol. Isolectin-B4 was purchased from Sigma-Aldrich LLC, and primary antibody of NG2 (ab50009) and secondary antibody against mouse (ab150115) were purchased from Abcam plc. Retinas were photographed using a confocal microscopy (Fluoview 1000, Olympus, Tokyo, Japan). In isolectin-B4 staining, the superficial vascular plexus (SVP) was presented with green, and the inner and outer deep vascular plexus (iDVP, oDVP) were yellow and purple, respectively. A comprehensive illustration of the retinal vasculature was generated and assembled using Adobe Photoshop and Adobe Illustrator (Adobe Systems, San Jose, CA, USA). In isolectin-B4 and NG2 double staining, vessels were shown as green and NG2 was red.

### 2.3. Immunohistochemistry (IHC)

The eyeballs were collected and embedded in paraffin. Sections (6 *μ*m) were cut sagittally, rehydrated, and incubated with TGF-beta1 primary antibody (ab92486, Abcam, Cambridge, MA, USA) overnight at 4°C. Immune complexes were detected with a goat anti-rabbit IgG (ab6721) secondary antibody. Finally, nucleus was stained with hematoxylin. Images were photographed with a light microscopy (Eclipse Ci, Nikon, NY, USA). To quantify the expression of TGF-beta1, color intensity of the TGF-beta1-positive regions was calculated using Image pro plus and plotted by GraphPad prism.

### 2.4. Electron Microscopy

Fresh retinas were isolated, and tissue blocks of the peripheral area were obtained. The tissue blocks were placed into 2% paraformaldehyde (PFA) and 0.05% glutaraldehyde at 4°C for 2-4 h and then washed in PBS three times, 15 min each. Afterwards, the tissue samples were fixed with 1% OsO4 in PBS for 2 h at room temperature and rinsed in PBS three times, 15 min each. Dehydration was conducted as follows: 50% ethanol for 15 min, 70% ethanol for 15 min, 80% ethanol for 15 min, 90% ethanol for 15 min, 95% ethanol for 15 min, two changes of 100% ethanol for 15 min, and two changes of acetone for 15 min. Infiltration was implemented as follows: 1:1 acetone: EMBed 812 for 2-4 h, 2:1 acetone: EMBed 812 overnight in a dessicator, and pure EMBed 812 for 5-8 h in a 37°C oven overnight. The retinas were then embedded in an oven at 60°C for 48 h, and 60 nm sections were obtained. The sections were then stained with uranyl acetate in pure ethanol for 15 min and rinsed with distilled water, followed by staining with lead citrate for 15 min and rinsing with distilled water. Finally, the retinas were air-dried overnight and observed with an electron microscopy (HT7700, HITACHI, Japan).

### 2.5. Western-Blot

Protein was extracted using the Protein Purification Kit (KeyGEN BioTECH, Nanjing, China), and the concentration was measured with the BCA protein assay (KeyGEN BioTECH). Protein was further denatured and prepared for electrophoresis. Afterwards, the protein was transferred onto PVDF membranes and incubated with anti-pSMAD2/3 (8828S, CST, MA, USA), anti-SMAD2/3 (8685, CST, MA, USA), anti-CD31 (ab222783), anti-Rho (ab155097), and anti-GAPDH (ab9485). The secondary antibodies were goat anti-rabbit (ab6721) and goat anti-mouse (ab6789) antibodies.

### 2.6. Statistical Analysis

The results were expressed as the means ± standard deviation (SD). To compare data between the OIR and Normoxia groups, an unpaired t-test was used. To compare data among the four groups, ANOVA was used, and differences between the groups were analyzed by the LSD t-test. All analyses were two-tailed and performed using SPSS 16.0 (IBM, NY, USA). Graphs were prepared using GraphPad Prism (San Diego, CA, USA). Significance was set at *∗*P<0.05.

## 3. Results

### 3.1. ODVP Reached the Edge of Retina on D14

**Figure 1:**
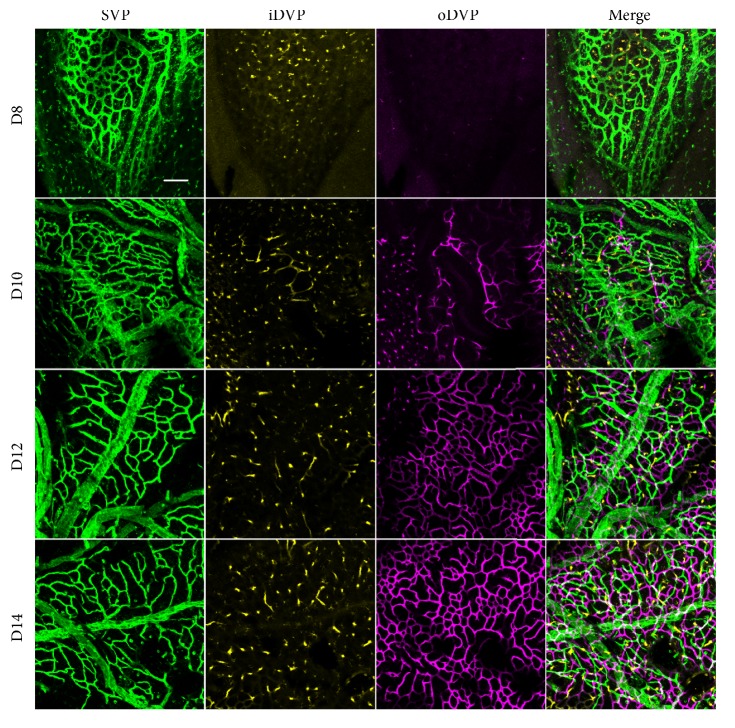
*Central retinal vasculatures development from D8 to D14*. From D8 to D14, SVP and iDVP were present in the central region and proliferated along time. In contrast, the oDVP was not visible in D8 retina. In the retina of D10, oDVP started to grow in the central region. Afterwards, the oDVP sprouted on D12 and proliferated on D14. SVP: superficial vascular plexus (green); iDVP: inner deep vascular plexus (yellow); oDVP: outer deep vascular plexus (purple). Taken together, the timeline of the normal development of central oDVP in newborn rats was from D8 to D14. Scale bar=50 *μ*m.

**Figure 2:**
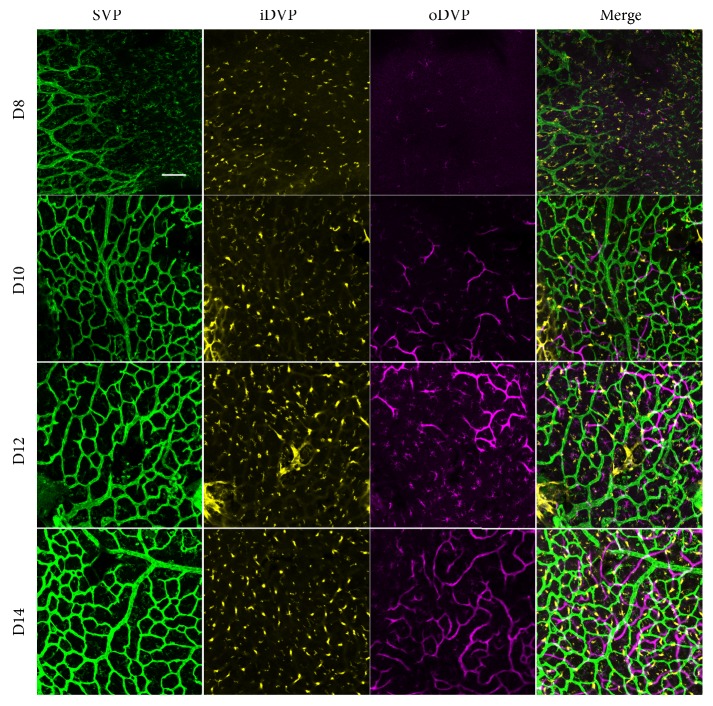
*Peripheral retinal vasculatures development from D8 to D14*. From D8 to D14, SVP and iDVP were present in the peripheral region and proliferated along time. In contrast, the oDVP was not present at D8. In the D10 retina, the oDVP emerged in the peripheral retina. Afterwards, the oDVP sprouted at D12 and reached the edge of retina on D14. Taken together, the timeline of the normal development of peripheral oDVP in newborn rats was from D8 to D14. Scale bar=50 *μ*m.

We explored the development of vasculatures, including the superficial vascular plexus (SVP, green), inner deep vascular plexus (iDVP, yellow), and outer deep vascular plexus (oDVP, purple). Figures 1 and 2 presented the central and peripheral areas, respectively. In Figures 1 and 2, the oDVP was not present in the central or peripheral areas on D8. On D10, the oDVP appeared in the central and peripheral areas. Afterwards, the oDVP continued to grow and finally reached the edge of retina on D14. Taken together, the timeline of the normal development of oDVP in newborn rats was from D8 to D14.

### 3.2. TGF-Beta1/SMAD2/3 Signaling Was Downregulated in the OIR Retinas

**Figure 3:**
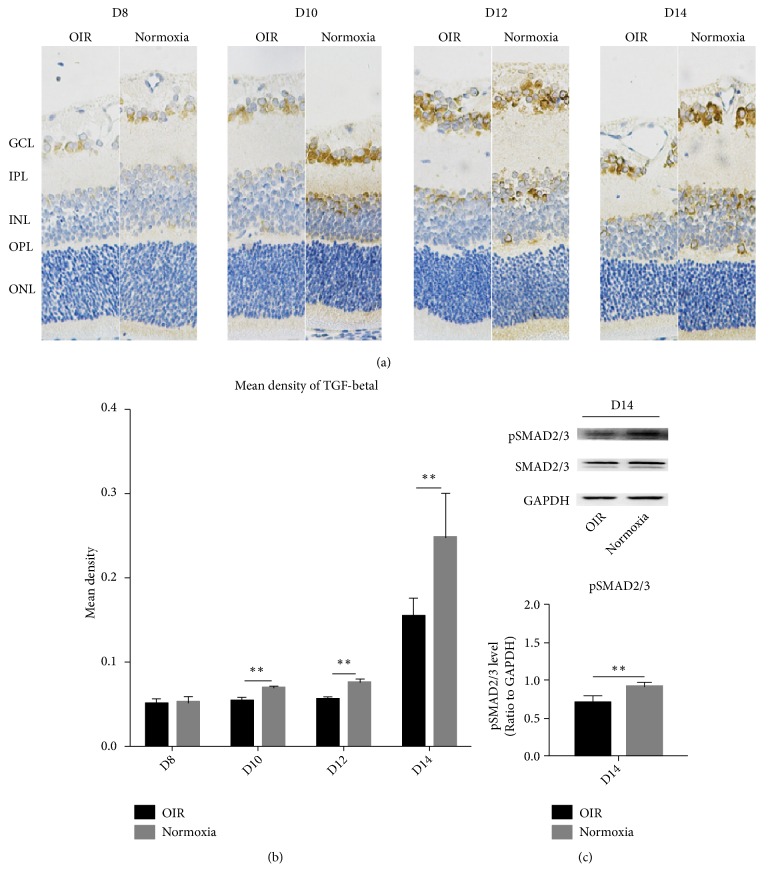
*Representative images of TGF-beta1 IHC and pSMAD2/3 western-blot in the OIR and Normoxia groups*. (a, b) showed the IHC and quantification of TGF-beta1 from D8 to D14 in the two groups. On D8, TGF-beta1 expression clustered in GCL and the expression was not significantly different. From D10 to D14, TGF-beta1 was significantly stronger in the Normoxia group, appearing in the IPL, INL, and OPL, and was absent from the OPL in the OIR group. As shown in (c), pSMAD2/3 was downregulated in OIR retinas on D14. SMAD2/3 were at similar levels in the two groups. GAPDH was used as a loading control. As shown in (c), the expression of pSMAD2/3 was significantly higher in the Normoxia group at D14. Data were shown as means±SD. GCL: ganglion cell layer, IPL: inner plexus layer, INL: inner nuclear layer, OPL: outer plexiform layer, ONL: outer nuclear layer (*∗∗*P<0.01).

To explore TGF-beta1 expression and location, IHC was performed. As shown in Figure 3(a), TGF-beta1 was predominantly expressed in the ganglion cell layer (GCL), inner nuclear layer (INL), inner plexiform layer (IPL), and outer plexiform layer (OPL) but was absent in the outer nuclear layer (ONL). At D8, TGF-beta1 was not significantly different between the two groups. However, from D10 to D14, TGF-beta1 expression was significantly higher in the Normoxia group (Figure 3(b)). In conclusion, during the development of oDVP (D8-14), TGF-beta1 was downregulated in the OIR retinas.

In western-blot of pSMAD2/3 of the whole retina (Figures 3(c) and 3(d)), it was downregulated in OIR retina on D14, indicating that the TGF-beta1/SMAD2/3 signaling pathway was compromised in the OIR model.

### 3.3. Characteristics of the Retinal Vasculatures in the OIR, OIR+LY364947, Normoxia, and Normoxia+LY364947 Groups

#### 3.3.1. CD31 Was Reduced by OIR Induction and Inhibitor Application

**Figure 4:**
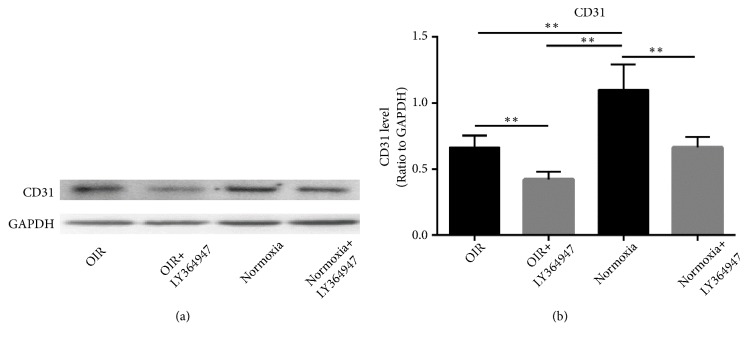
*OIR induction and inhibitor administration decreased CD31 expression*. (a, b) displayed the western-blot and quantification of CD31 in the four groups at D14. GAPDH was used as loading control. It was shown that CD31 reduced in the OIR group compared with the Normoxia group. Furthermore, LY364947 decreased the expression in the inhibitor groups compared with the noninhibitor counterparts (*∗∗*P<0.01).

CD31 was used as the marker of endothelial cells, the expression of CD31 (Figure 4) in the Normoxia group was greater than those in the OIR and OIR+LY364947 groups. In addition, the inhibitor compromised the expression in the OIR+LY364947 and Normoxia+LY364947 groups compared with those in their noninhibitor counterparts. The results indicated the negative role of OIR induction in early stage of retinal vascular development. Furthermore, the downregulation of TGF-beta1/SMAD2/3 pathway might participate in the obstruction of vascular development.

#### 3.3.2. Isolectin-B4 Staining Revealed Inhibited DVP Development in the OIR and Decreased DVP Density in LY364947 Groups

**Figure 5:**
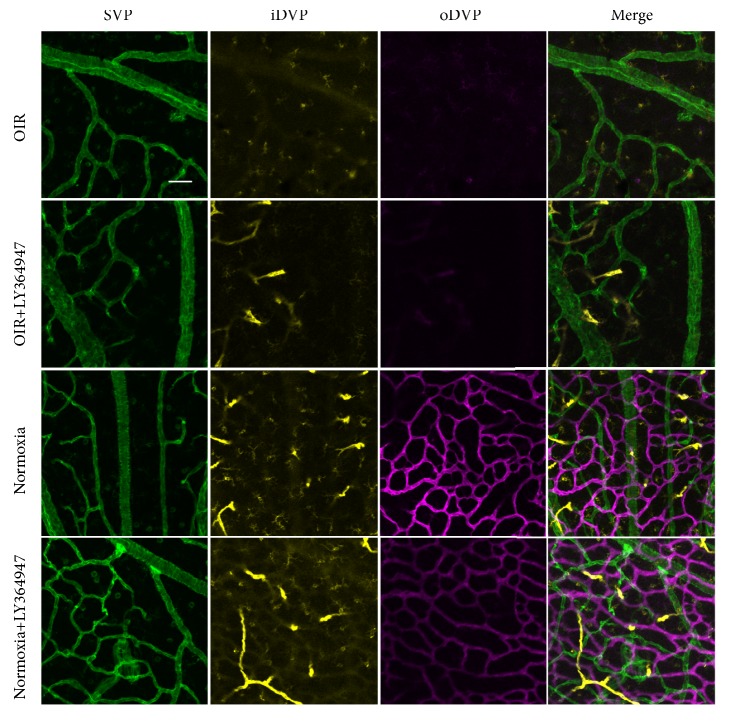
*Central retinal vasculatures in the four groups*. Flat-mount staining of isolectin-B4 in the central region in OIR, OIR+LY364947, Normoxia, and Normoxia+LY364947 groups at D14. Compared with retina from the Normoxia and Normoxia+LY364947 groups, retinas from the OIR and OIR+LY364947 groups showed attenuated growth of the iDVP and oDVP. Compared with the Normoxia group, oDVP showed decreased density and intensity in the Normoxia+LY364947 group (scale bar=25*μ*m).

**Figure 6:**
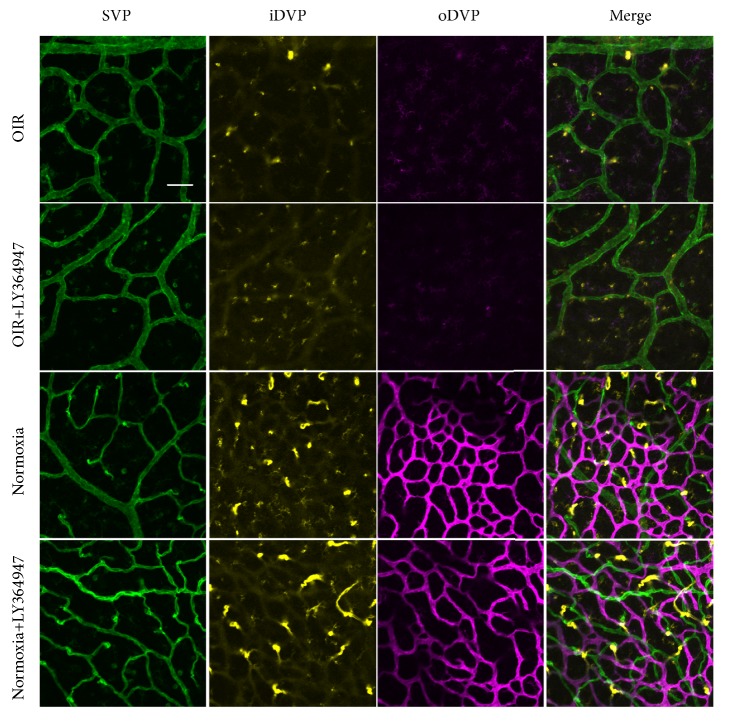
*Peripheral retinal vasculatures in the four groups*. Flat-mount staining of isolectin-B4 in the peripheral region in OIR, OIR+LY364947, Normoxia, and Normoxia+LY364947 groups at D14. Compared with retina from the Normoxia and Normoxia+LY364947 groups, retinas from the OIR and OIR+LY364947 groups showed attenuated growth of the iDVP and oDVP. Compared with the Normoxia group, oDVP showed decreased density and intensity in the Normoxia+LY364947 group (scale bar=25*μ*m).

As shown in Figure 5, central fields in the OIR or OIR+LY364947 retinas were deprived of DVP development as illustrated by absence of yellow and purple vasculatures. In contrast, both the Normoxia and Normoxia+LY364947 groups showed DVP development in the central region. Compared with the Normoxia group, oDVP showed decreased density and intensity in the Normoxia+LY364947 group. The results showed attenuated development of DVP after OIR induction and inhibitor application. Similar results were observed in the peripheral region in Figure 6.

**Figure 7:**
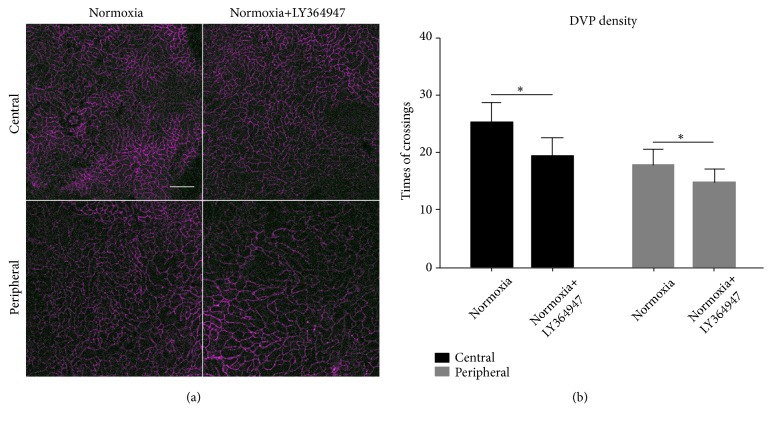
*ODVP density in the four groups*. (a) displayed the oDVP in the central and peripheral regions in the Normoxia and Normoxia+LY364947 groups at D14. Scale bar=100*μ*m. (b) was the quantification of vascular density (represented by times of crossings). Combining (a) and (b), it was shown that the inhibitor significantly reduced the densities in both the central and peripheral fields (*∗∗*P<0.01).

To further compare the difference of oDVP density in the Normoxia and Normoxia+LY364947 groups, pictures of oDVP were clustered and presented in Figure 7(a). Densities of oDVP were calculated by drawing vertical and horizontal lines on the pictures and counting the time of crossings between lines and vasculatures. The results were presented in Figure 7(b). In the Normoxia+LY364947 group, oDVP showed lower densities compared with the Normoxia group in both central and peripheral regions, indicating attenuated development of oDVP after inhibitor application.

#### 3.3.3. Pericyte Coverage Decreased in the OIR and LY364947 Groups

**Figure 8:**
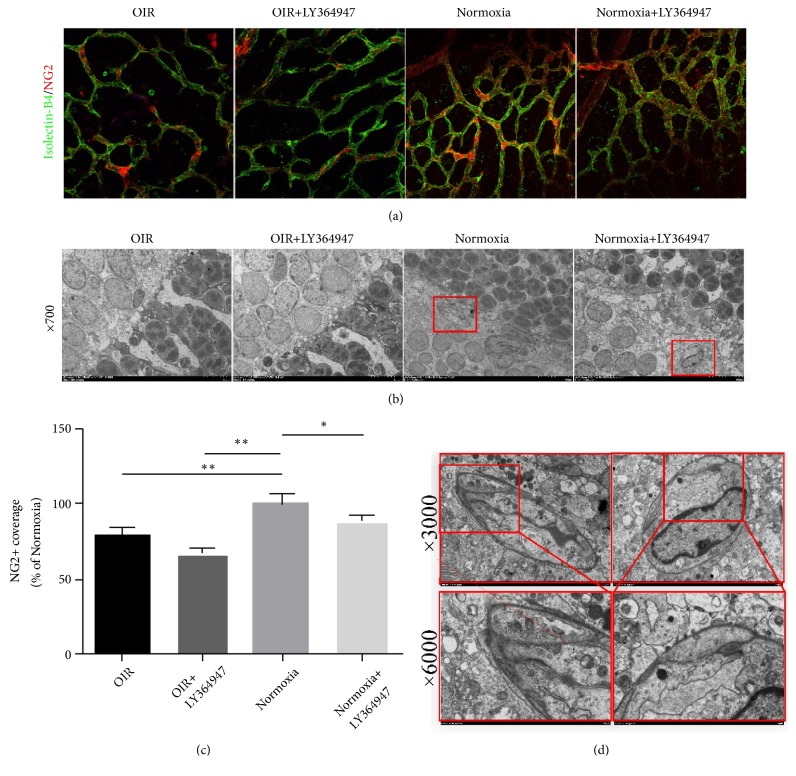
*Pericyte immunofluorescence and oDVP in electron microscopy*. (a) presented the flat-mount staining of isolectin-B4 and NG2 at D14. Pericyte reduced in the OIR group compared with the Normoxia group. LY364947 decreased pericyte coverage in the OIR+LY364947 and Normoxia+LY364947 groups compared with their noninhibitor counterparts (×400, scale bar=25*μ*m). (c) showed the quantification of NG2 coverage in those groups compared with the Normoxia group. (b, d) showed the electron microscopy pictures of the OPL regions in the four groups and magnified fields below of the latter two groups, elucidating the capillary (×3000) and pericyte (×6000), respectively. In the peripheral retina, the oDVP did not appear in the OPL region in the first two groups but were detectable in the Normoxia and Normoxia+LY364947 groups (×700, scale bar=10*μ*m; ×3000, scale bar=2*μ*m). In the enlarged fields (×6000, scale bar=1*μ*m), pericyte in the Normoxia+LY364947 group showed fewer organelles and vesicles than that in the Normoxia group.

In retinal flat-mount staining of Isolectin-B4 and NG2 (Figure 8(a)), pericyte coverage decreased in the OIR group compared with the Normoxia group. In addition, LY364947 further decreased NG2 staining in the OIR+LY364947 and Normoxia+LY364947 groups compared with their noninhibitor counterparts. The quantification was plotted in Figure 8(c).

As captured by the electron microscopy (Figure 8(b)), in the peripheral retina, the oDVP did not appear in the OIR and OIR+LY364947 groups, but it was detectable in the Normoxia and Normoxia+LY364947 groups. In enlarged fields (Figure 8(d)) of the latter two groups, pericytes in the Normoxia+LY364947 group showed fewer organelles and vesicles than those in the Normoxia group, indicating compromised pericyte development after inhibitor application.

### 3.4. Characteristics of Photoreceptors in the Four Groups

**Figure 9:**
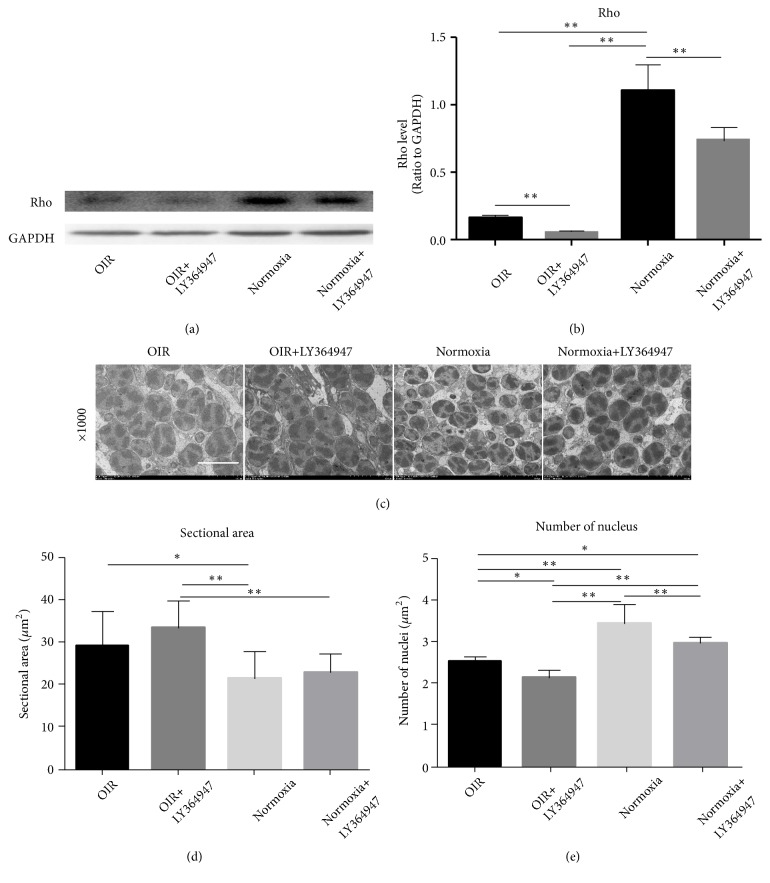
*Rho expression and photoreceptor phenotype*. The expression of Rho was presented in (a) with GAPDH as a loading control, and the quantification of Rho was displayed in (b). In western-blot, Rho in the Normoxia group was significantly higher than that in the OIR group, LY364947 decreased the expression in the OIR+LY364947 and Normoxia+LY364947 groups compared with their noninhibitor counterparts. (c) showed the representative images of photoreceptors at 1000×magnification (scale bar=10*μ*m). The average sectional area and number of nucleus were calculated and plotted in the graphs (d, e). The average sectional area was significantly larger in the OIR group compared with the Normoxia group (*∗*P<0.05); similarly, OIR induction also increased the area in OIR+LY364947 group compared with the Normoxia+LY364947 group. OIR together with inhibitor administration further expanded the gap, as shown by the comparison of the OIR+LY364947 group with the Normoxia group. In terms of the number of nucleus (c), it was decreased by OIR induction, inhibitor application, and the two combined, in comparison with OIR, Normoxia+ LY364947, and OIR+ LY364947 with Normoxia group, respectively; inhibitor application also decreased the number in the OIR+LY364947 compared with its noninhibitor counterpart; furthermore, OIR induction reduced the nucleus number in comparison of the OIR versus Normoxia+ LY364947 group and OIR+ LY364947 versus Normoxia+ LY364947 group. Data were shown as means±SD. (*∗*P<0.05, *∗∗*P<0.01).

In western-blot analysis, Rho, which was the biomarker of rod photoreceptor, decreased in the OIR and OIR+LY364947 groups compared with that in the Normoxia group. In addition, the inhibitor decreased Rho expression in both the OIR+LY364947 and Normoxia+LY364947 groups compared with those in their noninhibitor counterparts, indicating the negative role of inhibitor in photoreceptor development (Figures 9(a) and 9(b)).

As shown in Figure 9(c), the photoreceptor nucleus was enlarged in the OIR and OIR+LY364947 groups compared with those in the Normoxia and Normoxia+LY364947 groups. To measure the sectional area, the nucleus was simplified to an oval for calculation and the result was plotted in a bar graph (Figure 9(d)). The mean sectional area was significantly larger in the OIR group than that in the Normoxia group. Furthermore, the area in the OIR+LY364947 group was significantly larger than those in the Normoxia and Normoxia+LY364947 groups. Regarding nuclear number (Figures 9(c) and 9(e)), compared with the Normoxia group, it was decreased by OIR induction, inhibitor application, and the two combined as shown in the OIR, Normoxia+LY364947 and OIR+LY364947 groups. Combining the results above, OIR induction and inhibitor both participated in increasing the sectional area and decreasing the photoreceptor number, consequently playing a negative role in photoreceptor development.

The data used to support the findings of this study are available from the corresponding author upon request.

## 4. Discussion

**Figure 10:**
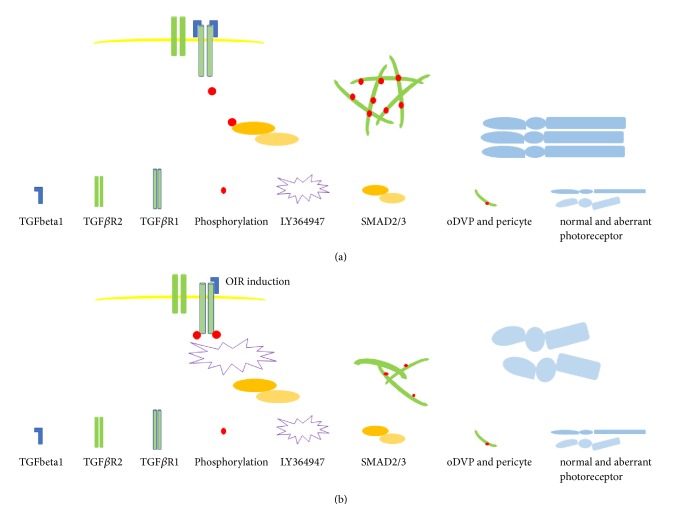
*Schematic representation of the study*. OIR induction or LY364947 inhibited TGF-beta1/SMAD2/3 signaling in different ways, caused oDVP (periyte) malformation, and exerted negative effects on (rod) photoreceptor development.

In the present study, we found that the oDVP started to grow on D10 and reached the edge of retina on D14. To the best of our knowledge, this is the first study illustrating the timing of oDVP development in newborn rats. Coincidentally, TGF-beta1 in the OIR group was downregulated from D10 to D14 and failed to express at the OPL region, where oDVP located. Based on previous study which reported the DVP malformation in the TGF-beta1 knock-out mice [11], these results implied the possible relationship between lower TGF-beta1 expression and oDVP malformation in OIR. In addition, pSMAD2/3 was downregulated in the OIR rats on D14, indicating reduced TGF-beta1/SMAD2/3 pathway signaling during oDVP development. Based on these findings, we administered a TGF-beta1/SMAD2/3 pathway inhibitor, LY364947, from D10 to D14 in both Normoxia and OIR rats. In the Normoxia+LY364947 retinas, total vascular retardation was indicated by CD31 downregulation; the development of the oDVP and pericyte was also blocked, as shown in the flat-mounting staining and electron microscopy in the OPL region; additionally, photoreceptor growth was found to be deteriorated according to the results of electron microscopy and decreased Rho expression. These results indicated that the TGF-beta1/SMAD2/3 pathway inhibitor mimicked the vascular retardation and photoreceptor malformation. Furthermore, in the OIR+LY364947 retinas, DVP and photoreceptor development were further compromised compared with the OIR retinas. Therefore, we concluded that the TGF-beta1/SMAD2/3 pathway participated in DVP (pericyte) formation and its downregulation partially contributes to DVP phenotype in OIR, exerting effects on photoreceptor growth (Figure 10).

The function of TGF-beta1 in vascularization is controversial. Studies exploring the effects of TGF-beta1 functions in vascularization often lead to conflicting conclusions [20–22]. Its essential role in cerebral vascular development was illustrated by selective deletion of the TGF-beta receptor 2 or Alk5 genes in endothelial cells, which resulted in embryonic lethality due to brain-specific vascular pathologies [23]. However, TGF-beta1 secreted from amniotic membrane-derived MSCs (AMSCs) suppressed the proliferation of endothelial cells under pathological conditions in vitro. In addition, in a mouse OIR model, AMSCs migrated into the retina and suppressed neovascularization via TGF-beta1 expression [24]. Therefore, the roles of TGF-beta1 are highly context-dependent.

Apart from the endothelial cells, the TGF-beta1/SMAD2/3 pathway has also been confirmed to have effects in pericyte growth [12]. Similarly, the pericyte was disturbed in the Normoxia+LY364947 group, as shown by electron microscopy and flat-mount staining in the present study.

Considering the physical contact and clinical association of oDVP and photoreceptors, the present study confirmed the effect of TGF-beta1 on photoreceptors possibly by modifying oDVP development. In previous studies, TGF-beta1 has been proven to have functions in various diseases related to neuronal disorders mainly by modulating microglial effects [25]. For example, TGF-beta1 was reported to be neuroprotective in Parkinson's disease by blocking microglial inflammatory responses via TGF-beta1/pSMAD2/3 signaling [26]. Similarly, TGF-beta1 modulates the microglial phenotype partly by decreasing microglial IL6 expression and promotes recovery after intracerebral hemorrhage [25]. In conclusion, other mechanisms such as inflammatory modulation and direct functions of TGF-beta1 in OIR warrant further exploration.

There are limitations in the present study. Apart from TGF-beta1/SMAD2/3 pathway in vascular development, noncanonical pathways have also been reported in vascularization but were not investigated in the present study. In addition, the reasons for the rather moderate phenotype of oDVP in the inhibitor groups might be that the dosage was comparatively low. Modifications will be made in further studies, and knock-down/knock-out animal models could be used in further study in order to rule out the interference.

## 5. Conclusions

The present study investigated the retinal oDVP and photoreceptor development and malformation following TGF-beta1/SMAD2/3 inhibition. We reported that inhibition of TGF-beta1 signaling in the retinal microenvironment of newborn rat induced the changes that largely mimic the phenotype of ROP in rat OIR model. The data also supported the potential role of oDVP malformation in photoreceptor damage, suggesting a possible therapeutic target to prevent photoreceptor damage.

## Data Availability

The data used to support the findings of this study are available from the corresponding author upon request.
